# Efficacy of an Emergency Cervical Cerclage Using Absorbable Monofilament Sutures

**DOI:** 10.1155/2018/4049792

**Published:** 2018-11-26

**Authors:** Yuka Sato, Nobuhiro Hidaka, Takahiro Nakano, Saki Kido, Masahiro Hachisuga, Yasuyuki Fujita, Kiyoko Kato

**Affiliations:** Department of Obstetrics and Gynecology, Graduate School of Medical Sciences, Kyushu University, Japan

## Abstract

**Introduction:**

Although nonabsorbable woven tape has been widely used for cervical cerclage, technical difficulties that can occur with an effaced cervix because of the thickness of the tape, and the risks of local infection are two major concerns. This study investigated perinatal outcomes of pregnancies involving an emergency cervical cerclage using absorbable monofilament polydioxanone sutures, which is a narrow thread and protects against bacterial infection.

**Materials and Methods:**

We performed a chart review of patients who underwent emergency McDonald cerclage with polydioxanone sutures at our institution between 2007 and 2015. Gestational age at delivery, duration between cerclage and delivery, and neonatal prognosis were evaluated as primary outcomes.

**Results:**

Among the 23 patients (18 singleton and five twin pregnancies) evaluated, ultrasound-indicated (progressive cervical length shortening) were eight (35%) and physical examination-indicated (fetal membranes that prolapsed into the vagina or dilated cervix) were 15 patients (65%). The median gestational age at cerclage was 22^+3^ weeks (range, 17^+5^ to 25^+3^ weeks). Postoperative spontaneous abortion occurred in only one patient. The median gestational age at delivery was 32^+5^ weeks (range, 20^+5^ to 40^+6^ weeks). Extremely preterm delivery before 28 weeks of gestation occurred in four (17%) cases. Full-term delivery was achieved in 10 (42%) cases. The duration between cerclage and delivery ranged from 5 to 136 days (median, 77 days). Except for one case of spontaneous abortion, all newborns survived till hospital discharge.

**Conclusions:**

Although our series included some patients at high risk for spontaneous abortion and preterm delivery, satisfactory prolongation and favorable neonatal outcomes were achieved for most patients by using absorbable monofilament sutures, thus suggesting the efficacy of this type of suture for emergency cervical cerclage.

## 1. Introduction

Cervical cerclage is an important method of preventing preterm delivery. Therefore, emergency cerclage is sometimes applied during the second trimester in women noted to have painless cervical dilation or progressive cervical shortening. Various studies regarding the efficacy of emergency cerclage for women at high risk have been published; however, the relative merits of the available suture materials have not been fully discussed. No randomized trials comparing different types of cerclage suture materials have been performed. A study investigating perioperative management strategies [1] indicated that nonabsorbable sutures are recommended. Nonabsorbable woven tape is widely used for cervical cerclage worldwide; however, technical difficulties that can occur with an effaced cervix because of the thickness of the tape, and the risk of local infection are two possible major disadvantages of nonabsorbable woven tape. To resolve these problems, in 2007, we established a new approach to cerclage by using absorbable monofilament polydioxanone sutures. The absorbable monofilament polydioxanone suture is a narrow thread, and protects against bacterial infection. This study aimed to investigate perinatal outcomes of pregnancies involving an emergency cervical cerclage using absorbable material and to assess the efficacy of and problems associated with this type of suture.

## 2. Materials and Methods

A retrospective cohort study was conducted involving all women who underwent emergency cervical cerclage with polydioxanone sutures at our institution between 2007 and 2015. The Kyushu University Hospital Ethical Review Board approved the study (approval number 29-174), and all work was conducted in accordance with the Declaration of Helsinki (1964). Medical charts were reviewed, and gestational age at delivery, time from cerclage to delivery, and neonatal survival were considered the primary outcomes for this study. In addition to these data, the following were recorded as secondary outcomes: intraoperative rupture of membranes, postoperative preterm premature rupture of membranes (pPROM), clinical chorioamnionitis (CAM), and neonatal grade 3 or grade 4 intraventricular hemorrhage (IVH). Data are reported as median and range when data were not normally distributed.

Our institutional indications for emergency cerclage included no significant uterine contractions or pPROM, the absence of clinical CAM as evidenced by axillary temperature >38.0°C, serum white blood cell count more than 15×10^9^, and C-reactive protein level more than 2.0 mg/dL. Ultrasound-indicated cerclage was performed for patients exhibiting progressive cervical length shortening; physical examination-indicated cerclage was performed for patients with fetal membranes that prolapsed into the vagina or dilated cervix. Preoperative microbiological analyses were performed using vaginal swabs, but amniotic fluid cultures were not obtained because the results were not available at the time of emergency cerclage. A fetal anatomic survey was performed, and fetal anomalies incompatible with life were ruled out. The cerclage procedure using the McDonald method was performed under spinal anesthesia. An absorbable monofilament suture, 0 PDS PLUS (Ethicon Inc., Sommerville, NJ), was used to suture the cervix. When necessary, moist sterile gauze was used with gentle pressure to place the membranes back into the uterus and to push the membranes to allow suturing. Prophylactic intravenous ritodrine hydrochloride or magnesium sulphate was administered for tocolysis during postoperative care. Prophylactic broad-spectrum antibiotics were also administered before, during, and after cerclage. Antenatal corticosteroids were used when the physician deemed preterm delivery to be imminent before 34 weeks. The suture material was removed for all women who underwent labor, had ruptured membranes, or developed clinical CAM, and those who reached 36 weeks of gestation.

## 3. Results

During the study period, 23 patients met the inclusion criteria.  Table 1 shows the baseline maternal characteristics. Of 23 pregnancies, 18 were singleton and five were twin pregnancies. The median patient age was 33 years (range, 18–41 years). Twelve women were primiparous. Six women had a history of preterm birth.

Table 2 shows the gestational age at cerclage and indications. The median gestational age at cerclage was 22^+3^ weeks (17^+5^ to 25^+3^). Among the 23 pregnancies, indications for cerclage were ultrasound-indicated for 8 (35%) and physical examination-indicated for 15 (65%). Among the physical examination-indicated cases, dilatation of the cervix without uterine contractions occurred in 10 (43%) and fetal membranes prolapsed into the vagina at the time of cerclage in five (22%) patients. Membrane rupture during cerclage was not noted, and the cerclage procedure was completed in all 23 cases.

**Figure 1:**
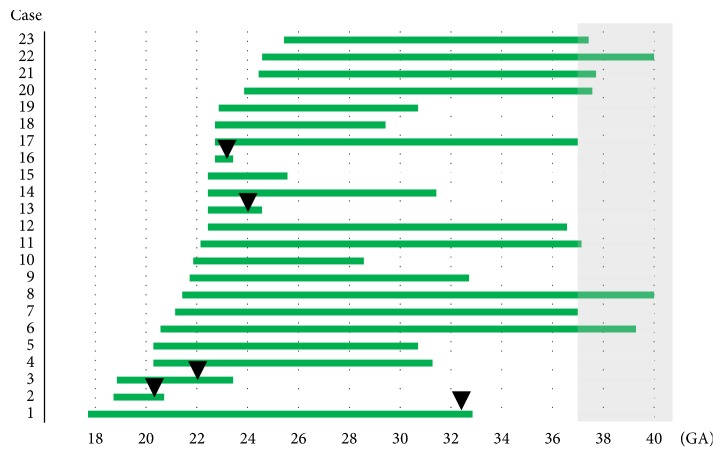
**Duration between cerclage and delivery.** GA, gestational age (weeks). *▾* indicates GA at preterm rupture of membranes.

Table 3 shows pregnancy and perinatal outcomes. Postoperative pPROM occurred in five patients. The median gestational age at pPROM was 23^+3^ weeks (range, 20^+5^ to 32^+6^) for these five patients, and the median time lag between cerclage and pPROM was 14 days (range, 5–106 days). Spontaneous labor pain could not be inhibited during the previable period (after pPROM at 20 weeks of gestation), resulting in spontaneous abortion for one patient. The median gestational age at delivery among the 23 patients was 32^+5^ weeks (range, 20^+5^ to 40^+6^ weeks). Extremely preterm delivery prior to 28 weeks of gestation occurred for four (17%) patients, and full-term delivery was achieved by nine (39%) patients. The duration between cerclage and delivery (Figure 1) ranged from 5 to 136 days (median, 77 days). Among 11 cases for which pathological examination of the placenta was performed, CAM was demonstrated in six cases. In all cases, 0 PDS PLUS had already become brittle, and thus, the suture removal was technically easy.

Table 4 shows the outcomes of 27 live-born neonates, except for one case of spontaneous abortion. All newborns survived to hospital discharge. Neonatal death and infantile death were not encountered. Only one newborn had grade 3 IVH. The mother had a history of spontaneous abortion at 21 weeks of gestation and underwent emergency cerclage at 18 weeks of gestation because of dilatation of the cervix without any uterine contractions. Despite postoperative administration of tocolytic agents, including ritodrine hydrochloride and magnesium sulphate, spontaneous labor pain occurred, resulting in extremely preterm birth at 23 weeks, 3 days of gestation. The infant weighed 556 g, had Apgar scores of 2 and 4, and developed grade 3 IVH. The newborn was treated in the neonatal intensive care unit and discharged at 136 days of life. Currently, the child is 2 years old and is being followed up at our hospital. He was diagnosed as having an intellectual disability.

Perinatal outcomes are presented in Table 5, based on the indications: ultrasound-indicated versus physical examination-indicated. The incidence of pPROM, preterm delivery, and duration between cerclage and delivery were not significantly different between the two groups; however, the incidence of pPROM appeared higher in the physical examination-indicated group than that in the ultrasound-indicated group (26.7% vs 12.5%).

## 4. Discussion

The present cohort study of patients who underwent McDonald cervical cerclage with polydioxanone sutures showed that a satisfactorily prolonged pregnancy period was achieved. All newborns except for one (spontaneous abortion) survived to hospital discharge.

Shirodkar first reported about transvaginal cerclage during pregnancy [2]. The most commonly used procedure is McDonald cerclage [3]. The efficacy of emergency cerclage for prevention of preterm delivery for high-risk women with a short cervix has been proven by previous investigators [1, 4, 5]; therefore, cervical cerclage is an important procedure for preventing preterm delivery. However, the choice of suture material has not been sufficiently discussed until now. In the first study of cervical cerclage by Shirodkar [2], human fascia lata was used. Since then, several materials have been used for cerclage [1, 3]. The Royal College of Obstetricians and Gynaecologists Green-top Guideline No. 6 suggests that the choice of suture material should be determined at the discretion of the surgeon [6]; however, favorable suture materials were not mentioned in detail in that guideline. According to the Practice Bulletin of the American College of Obstetricians and Gynecologists, nonresorbable material is usually inserted during the McDonald procedure [7]. In clinical practice, the most commonly used materials are Mersilene™ tape [5] and nonabsorbable monofilament [1, 8]. Although nonabsorbable woven tape has been widely used for cervical cerclage, technical difficulties exist. Furthermore, the relative advantages of the available suture materials have not been fully assessed. Berghella et al. compared the ultrasound-indicated cerclage efficacy among the two types of nonabsorbable suture materials, braided polyester thread (Mersilene™ or Ethibond™) and Mersilene™ 5-mm tape, and suggested that these two suture materials have similar effects [9]. Bernard et al. performed a similar comparison involving women with a dilated cervix during the second trimester and reported that the use of braided sutures was associated with improved neonatal survival and prevention of preterm birth before 28 weeks of gestation [10]. Abdelhak et al. reported the first comparison of delayed absorbable sutures and nonabsorbable sutures for cervical cerclage after a retrospective study of 18 patients who underwent McDonald cerclage [11]. They compared the adverse outcomes between the two groups (14 patients treated with nonabsorbable material and four patients treated with absorbable material) and concluded that absorbable suture material might be a reasonable alternative because of the added benefit of spontaneous degradation instead of surgical removal. Nevertheless, there are only a few reports assessing the efficacy of cerclage using absorbable materials. Yorifuji et al. recently investigated the effectiveness of emergency cerclage using delayed absorbable monofilament material in six patients [12]. The mean gestational age at cerclage was 23.0 ± 0.9 weeks, and the average duration between cerclage and delivery was 13.4 ± 2.1 weeks. One limitation of the study by Yorifuji et al. was the small sample size; however, our data were based on 23 patients. Our treatment results were similar to those of Yorifuji et al., and they support the usefulness of absorbable monofilament sutures for emergency cerclage.

The absorbable monofilament suture, PDS PLUS (Ethicon Inc.) [13], retains 60% of its original tension strength for 6 weeks. However, our series has shown that the median duration between cerclage and delivery was more than 6 weeks. We speculated that not only the tension strength but also the scar tissue of the cervix after cerclage prevented cervical dilatation and contributed to the prolongation of pregnancy. Considering this, the nonabsorbability of the suture material may not be essential for sufficient prolongation of the gestational period.

This study had some limitations. First, it had a retrospective study design. Therefore, the cases that were judged as contraindicated for cerclage due to extreme technical difficulties by the attending physicians were excluded from the study analysis. This might have contributed to the better cerclage success rate. Second, this study was not a comparative analysis with nonabsorbable sutures; instead, it was a one-arm study. Therefore, further research is needed to determine the superior material.

## 5. Conclusions

The sample size included in our study was larger than that of previous studies investigating perinatal outcomes after emergency cerclage using absorbable materials. Although the study population included women at comparatively high risk for spontaneous abortion and preterm delivery and those with fetal membranes that had prolapsed into the vagina (22%), perinatal outcomes were satisfactory. Postoperative spontaneous abortion occurred for only one patient, and full-term delivery was achieved by 39%. Furthermore, except for one case of spontaneous abortion, all newborns survived to hospital discharge. Therefore, these satisfactory outcomes suggest that absorbable monofilament sutures can be used for emergency cerclage.

## Figures and Tables

**Table 1:** Baseline maternal characteristics.

Pregnancies, n	23

Singleton/twin, n	18/5

Maternal age, years	33 (range, 18–41)

Primiparous, n (%)	12 (50%)

Prior preterm delivery, n (%)	6 (25%)

**Table 2:** Surgical details.

Pregnancies, n	23

GA at cerclage, weeks	22^+3^ (17^+5^ to 25^+3^)

Indication	

Ultrasound-indicated, n (%)	8 (35%)

Cervical length, mm	8 (5-14)

Physical examination-indicated, n (%)	17 (65%)

Dilatation of cervix, n	10

Prolapsed membranes, n	5

ROM during cerclage, n (%)	0 (0%)

Surgery completed, n (%)	23 (100%)

GA, gestational age; ROM, rupture of membranes.

**Table 3:** Pregnancy and perinatal outcomes.

Pregnancies, n	23

pPROM, n (%)	5 (22%)

GA at pPROM, weeks	23^+3^ (20^+5^ to 32^+6^)

Clinical CAM, n (%)	2 (9%)

GA at delivery, weeks	32^+5^ (20^+5^ to 40^+6^)

<22 weeks, n (%)	1 (4%)

<28 weeks, n (%)	4 (17%)

≥28 weeks to <34 weeks, n (%)	8 (35%)

≥34 weeks to <37 weeks, n (%)	1 (4%)

≥37 weeks, n (%)	9 (39%)

Duration between cerclage and delivery, days	77 (5–136)

GA, gestational age; ROM, rupture of membranes; pPROM, preterm premature rupture of membranes; CAM, chorioamnionitis.

**Table 4:** Outcomes of live-born neonates.

Neonates, n	27

Outcome at discharge, n (%)	

Alive	27 (100%)

Not alive	0 (0%)

Birth weight, g	1915 (546–3600)

LBW (<2500 g), n (%)	20 (74%)

IVH grade 3 or 4, n (%)	1 (4%)

LBW, low birth weight; IVH, intraventricular hemorrhage.

**Table 5:** Perinatal outcome, by indication for cerclage.

Indication	Ultrasound	Physical examination
	N=15	N=8
GA at cerclage, weeks	21^+5^ (17^+5^ to 24^+3^)	22^+4^ (22^+3^ to 25^+3^)

pPROM, n	4/15	1/8

GA at pPROM, weeks	22^+6^ (20^+5^ to 32^+6^)	24^+4^

GA at delivery, weeks	32^+6^ (20^+5^ to 40^+6^)	31^+1^ (24^+4^ to 40^+0^)

<28 weeks, n	2/15	3/8

≥28 weeks to <34 weeks, n	3/15	5/8

≥37 weeks, n	3/15	6/8

Duration between cerclage and delivery, days	93 (5-136)	68 (15-108)

GA, gestational age; pPROM, preterm premature rupture of membranes.

## Data Availability

The data used to support the findings of this study are available from the corresponding author upon request.
